# Zolpidem Administration and Risk of Hepatocellular Carcinoma: A Case-Control Study in Taiwan

**DOI:** 10.3389/fphar.2017.00767

**Published:** 2017-10-23

**Authors:** Shih-Wei Lai, Cheng-Li Lin, Kuan-Fu Liao

**Affiliations:** ^1^College of Medicine, China Medical University, Taichung, Taiwan; ^2^Department of Family Medicine, China Medical University Hospital, Taichung, Taiwan; ^3^Management Office for Health Data, China Medical University Hospital, Taichung, Taiwan; ^4^College of Medicine, Tzu Chi University, Hualien, Taiwan; ^5^Department of Internal Medicine, Taichung Tzu Chi General Hospital, Taichung, Taiwan

**Keywords:** hepatocellular carcinoma, zolpidem, Taiwan, National Health Insurance Program

## Abstract

**Background/Objectives:** Previous studies showed that zolpidem use could be associated with increased cancer risk, but the role of zolpidem on hepatocellular carcinoma (HCC) risk remains undetermined. The study purpose was to examine the association between HCC risk and zolpidem use in Taiwan.

**Methods:** Using the database from the Taiwan National Health Insurance Program, we designed a case-control study which consisted of 77986 subjects aged 20 years or older with newly diagnosed HCC as the case group, and 77986 subjects without HCC as the control group, from 2000 to 2011. Ever use of zolpidem was defined as a subject who had at least a prescription for zolpidem before the index date. Never use was defined as a subject who did not have a prescription for zolpidem before the index date. The association between HCC risk and zolpidem use was determined by the odds ratio (OR) and 95% confidence interval (CI) in a multivariable logistic regression model.

**Results:** After adjustment for confounding factors, the adjusted OR of HCC was 1.05 (95% CI 0.97, 1.13) for subjects with ever use of zolpidem, compared with never use of zolpidem. The adjusted OR of HCC was 1.01 for subjects with increasing cumulative duration of zolpidem use for every 1 year (95% CI 0.99, 1.03), compared with never use of zolpidem.

**Conclusion:** There is no significant association between HCC risk and zolpidem use. There is no duration-dependent effect of zolpidem use on HCC risk.

## Introduction

Hepatocellular carcinoma (HCC) ranked the sixth most common cancer (854260 new cases, 4.9% of the total new cancer cases) and ranked the fourth leading cause of cancer deaths (810000 deaths, 9.3% of the total cancer deaths) globally in 2015 (Fitzmaurice et al., 2017). HCC ranked the second leading cause of cancer deaths in Taiwan in 2016 (8353 deaths, 17.5% of the total cancer deaths) (Ministry of Health and Welfare, Taiwan, 2017a).

Zolpidem is a non-benzodiazepine sedative-hypnotic medication commonly used to treat insomnia. To date, no *in vitro* study examines zolpidem use on cancer risk. Only few epidemiologic studies and review articles showed that zolpidem use could be associated with increased cancer risk (Kripke, 2008, 2016; Kripke et al., 2012; Sivertsen et al., 2015). Although drug-induced liver injury has been extensively studied, (Corsini et al., 2012; Hamilton et al., 2016) the case of zolpidem-related liver injury has never been reported and the toxic effect of chronic use of zolpidem on the liver has not been studied. Given that zolpidem is the most frequently prescribed non-benzodiazepine sedative-hypnotic medication in Taiwan, (Su et al., 2002; Hsiao et al., 2013) and HCC is highly prevalent in Taiwan, we make a rational hypothesis that zolpidem use is associated with HCC risk. If the association actually exists, clinicians should be more cautious of zolpidem use. Therefore, we designed a retrospective, population-based, case-control study using the database from the Taiwan National Health Insurance Program to examine whether there is an association between HCC risk and zolpidem use.

## Materials and Methods

### Data Sources

Taiwan is an independent country with more than 23 million persons (Chan et al., 2016; Chang and Yu, 2016; Chang et al., 2016; Hsieh et al., 2016; Liang et al., 2017; Liao et al., 2017b; Wen and Yin, 2017; Wu et al., 2017; Yang J.S. et al., 2017; Yang M.D. et al., 2017).

We designed a retrospective, population-based, case-control study using the database from the Taiwan National Health Insurance Program which has covered 99.6% of 23 million persons living in Taiwan in 2015 (Ministry of Health and Welfare, Taiwan, 2017b). The details of the insurance program can be found in previous studies (Lai et al., 2010, 2011; Chen et al., 2016; Tsai et al., 2016). The study was approved by the Research Ethics Committee of China Medical University and Hospital in Taiwan (CMUH-104-REC2-115).

### Inclusion Criteria

Based on the International Classification of Diseases 9th Revision-Clinical Modification (ICD-9 codes), we defined the cases as subjects aged 20 years or older who were newly diagnosed with HCC (ICD-9 codes 155, 155.0, and 155.2) from 2000 to 2011. We defined the index date for each case as the date of diagnosing HCC. For each case of HCC, we randomly selected one subject without HCC as the control. The HCC cases and the controls were frequency matched by sex, age (within 5 years) and the year of index date.

### Exclusion Criteria

Subjects with other cancers (ICD-9 codes 140–208) before the index date were excluded from the study.

### Comorbidities Studied

The comorbidities before the index date potentially associated with HCC risk were alcohol-related disease, cardiovascular disease, chronic kidney disease, chronic obstructive pulmonary disease, diabetes mellitus, hyperlipidemia, hypertension, as well as chronic liver disease including cirrhosis, hepatitis B infection, hepatitis C infection, and other chronic hepatitis. All comorbidities were diagnosed based on ICD-9 codes. The diagnosis accuracy of comorbidities was well discussed in previous studies (Lai et al., 2012, 2013; Liao et al., 2016, 2017a; Chu et al., 2017; Lin et al., 2017).

### Definition of Medication Exposure

History of prescriptions for zolpidem and benzodiazepines in Taiwan was included. To minimize a possible confounding effect, subjects whose cumulative duration of zolpidem use was <12 months were excluded from the study. Therefore, only subjects whose cumulative duration of zolpidem use was ≥12 months were included. The definition of medication exposure was adapted from previous studies (Cheng et al., 2017; Lai et al., 2017; Liao et al., 2017c,d). Subjects who did not have a prescription for medications studied were classified as never use. Subjects who ever had a prescription for medications studied were classified as ever use.

### Statistical Analysis

The differences in sex, age, zolpidem use, benzodiazepines use, and comorbidities between the HCC cases and the controls were compared by using the Chi-square test for categorized variables and the *t*-test for continuous variables. Variables found to be statistically significant in a univariable logistic regression model were further included in a multivariable logistic regression model. The odds ratio (OR) and 95% confidence interval were used to estimate the HCC risk associated with zolpidem use. A probability value of <0.05 was classified as statistical significance (SAS software version 9.2, SAS Institute Inc., Cary, NC, United States).

## Results

### Basic Data of the Study Population

Totally, 77986 cases with HCC and 77986 controls without HCC were included. **Table 1** presents the basic data between the HCC cases and the controls. The HCC cases and the controls had similar proportions of sex and age. The mean ages (standard deviation) were 61.7 (12.4) years in HCC cases and 61.6 (12.5) years in controls, without statistical significance (*t*-test, *P* = 0.06). The mean durations of exposure to zolpidem (standard deviation) were 3.06 (2.38) years in HCC cases and 3.03 (3.74) years in controls, without statistical significance (*t*-test, *P* = 0.71).

**Table 1:** Basic data between cases with hepatocellular carcinoma and controls.

	Controls *N* = 77986		Cases *N* = 77986		
			
Variable	*n*	(%)	*n*	(%)	*P*-value^∗^
Sex					0.98
Female	19537	(25.1)	19541	(25.1)	
Male	58449	(75.0)	58445	(74.9)	
**Age group (years)**					0.99
20–39	4228	(5.4)	4232	(5.4)	
40–64	39384	(50.5)	39383	(50.5)	
65–84	34374	(44.1)	34371	(44.1)	
			
Age (years), mean ± standard deviation^†^	61.6 ± 12.5		61.7 ± 12.4		0.06
			
Zolpidem use	2588	(3.32)	4207	(5.39)	<0.001
			
Duration of exposure to zolpidem (years), mean ± standard deviation ^†^	3.03 ± 3.74		3.06 ± 2.38		0.71
			
Benzodiazepines use	52596	(67.4)	62321	(79.9)	<0.001
**Comorbidities before index date**					
Alcohol-related disease	2961	(3.80)	11331	(14.5)	<0.001
Cardiovascular disease	22404	(28.7)	22137	(28.4)	0.13
Chronic kidney disease	4454	(5.71)	6778	(8.69)	<0.001
Chronic liver disease	10769	(13.8)	65490	(84.0)	<0.001
Chronic obstructive pulmonary disease	13940	(17.9)	14538	(18.6)	<0.001
Diabetes mellitus	10735	(13.8)	18652	(23.9)	<0.001
Hyperlipidemia	19966	(25.6)	14164	(18.2)	<0.001
Hypertension	33735	(43.3)	34595	(44.4)	<0.001


*Data are presented as the number of subjects in each group with percentages given in parentheses. ^∗^Chi-square test, and ^†^*t*-test comparing cases with hepatocellular carcinoma and controls.*

The HCC cases had higher proportions of zolpidem use (5.39% vs. 3.32%), benzodiazepines use (79.9% vs. 67.4%), alcohol-related disease (14.5% vs. 3.8%), chronic kidney disease (8.69% vs. 5.71%), chronic liver disease (84.0% vs. 13.8%), chronic obstructive pulmonary disease (18.6% vs. 17.9%), diabetes mellitus (23.9% vs. 13.8%), and hypertension (44.4% vs. 43.3%) than the controls, with statistical significance (Chi-square test, *P* < 0.001 for all).

### Association of Hepatocellular Carcinoma with Zolpidem Use, Benzodiazepines Use, and Comorbidities

After adjustment for confounding factors, the multivariable logistic regression model presented that the adjusted OR of HCC was 1.05 (95% CI 0.97, 1.13) for subjects with ever use of zolpidem, compared with those with never use of zolpidem. Other factors significantly associated with increased OR of HCC were benzodiazepines use (adjusted OR 1.50, 95% CI 1.45, 2.44), alcohol-related disease (adjusted OR 2.31, 95% CI 2.18, 2.44), chronic kidney disease (adjusted OR 1.21, 95% CI 1.14, 1.28), chronic liver disease (adjusted OR 34.2, 95% CI 33.1, 35.2), and diabetes mellitus (adjusted OR 1.93, 95% CI 1.85, 2.00) (**Table 2**).

**Table 2:** Odds ratio and 95% confidence interval of hepatocellular carcinoma associated with zolpidem use, benzodiazepines use, and comorbidities by multivariable logistical regression model.

	Crude		Adjusted^†^	
		
Variable	OR	(95% CI)	OR	(95% CI)
Sex (male vs. female)	1.00	(0.98, 1.02)	–	–
Age (per one year)	1.00	(0.99, 1.00)	–	–
Zolpidem use (never use as a reference)	1.66	(1.58, 1.75)	1.05	(0.97, 1.13)
Benzodiazepines use (never use as a reference)	1.92	(1.88, 1.97)	1.50	(1.45, 2.44)
Comorbidities before index date (yes vs. no)				
Alcohol-related disease	4.31	(4.13, 4.49)	2.31	(2.18, 2.44)
Cardiovascular disease	0.98	(0.96, 1.01)	–	–
Chronic kidney disease	1.57	(1.51, 1.63)	1.21	(1.14, 1.28)
Chronic liver disease	32.7	(31.8, 33.6)	34.2	(33.1, 35.2)
Chronic obstructive pulmonary disease	1.05	(1.03, 1.08)	0.85	(0.82, 0.88)
Diabetes mellitus	1.97	(1.92, 2.02)	1.93	(1.85, 2.00)
Hyperlipidemia	0.65	(0.63, 0.66)	0.28	(0.27, 0.29)
Hypertension	1.05	(1.03, 1.07)	0.93	(0.90, 0.96)


*^†^Variables found to be statistically significant in a univariable logistic regression model were further included in a multivariable logistic regression model. Adjustment for benzodiazepines use, alcohol-related disease, chronic kidney disease, chronic liver disease, chronic obstructive pulmonary disease, diabetes mellitus, hyperlipidemia, and hypertension.*

### Association of Hepatocellular Carcinoma with Cumulative duration of Zolpidem Use

We examined whether there was an association between cumulative duration of zolpidem use and HCC risk in **Table 3**. The sub-analysis presented that the adjusted OR of HCC was 1.01 for subjects with increasing cumulative duration of zolpidem use for every 1 year (95% CI 0.99, 1.03), compared with those with never use of zolpidem. It seemed that there was no duration-dependent effect of zolpidem use on HCC risk.

**Table 3:** The risk of hepatocellular carcinoma associated with cumulative duration of zolpidem use.

Variable	Case number/control number	Crude OR	(95% CI)	Adjusted OR^†^	(95% CI)
Never use of zolpidem as a reference	73779/75398	1.00	(reference)	1.00	(reference)
Cumulative duration of zolpidem use (increase in duration for every 1 year)	4207/2588	1.11	(1.10, 1.13)	1.01	(0.99, 1.03)


*^†^Variables found to be statistically significant in a univariable logistic regression model were further included in a multivariable logistic regression model. Adjustment for benzodiazepines use, alcohol-related disease, chronic kidney disease, chronic liver disease, chronic obstructive pulmonary disease, diabetes mellitus, hyperlipidemia, and hypertension.*

## Discussion

In this case-control study, we did not note a significant association between HCC risk and zolpidem use (**Table 2**). We did not note a duration-dependent effect between HCC risk and zolpidem use (**Table 3**). It seems that there are inconsistent results about cancer risk and zolpidem use between our study and other previous studies (Kripke, 2008; Kripke et al., 2012; Sivertsen et al., 2015). These previous studies did not adjust for HCC-related risk factors. In our case-control study, risk factors for HCC including alcohol-related disease, cirrhosis, hepatitis B infection, hepatitis C infection, and diabetes mellitus, (Lai et al., 2012) have been included for adjustment. Therefore, the confounding effects by these risk factors might be minimized in our study. That is why there is a difference among these results. The hypothesis that zolpidem use is associated with HCC risk is not convinced at present. It suggests that not all cancers have an association with zolpidem use. More research is needed to examine the risk of individual cancer and zolpidem use after adjustment for relevant confounding factors.

Some points need to be discussed. First, an observational case-control study cannot provide a pathophysiological answer for the association between HCC risk and zolpidem use. To date, no *in vitro* study examines zolpidem use on cancer risk. The case of zolpidem-related liver injury has never been reported and the toxic effect of chronic use of zolpidem on the liver has not been studied. Overall, this is an interesting and novel study in that it attempts to examine the issue for which little evidence is available. Second, HCC is a chronic disease. It is not appropriate to make a link between HCC risk and cumulative duration of zolpidem use <12 months. That is why subjects with cumulative duration of zolpidem use <12 months were excluded. Only subjects with cumulative duration of zolpidem use ≥12 months were included. Third, due to alpha-fetoprotein not being recorded in the database, we could not include this parameter for analysis. Fourth, we had access to a very useful database from the Taiwan National Health Insurance Program that has contributed to much epidemiological research in the population living in Taiwan. This strength is more convincing to the readers.

## Conclusion

There is no significant association between HCC risk and zolpidem use. There is no duration-dependent effect of zolpidem use on HCC risk. Further studies are needed to confirm our findings.

## Author Contributions

S-WL planned and conducted this study. He contributed to the conception of the article, initiated the draft of the article, and revised the article. C-LL conducted the data analysis and reviewed the article. K-FL planned and conducted this study. He participated in the data interpretation and revised the article.

## Conflict of Interest Statement

The authors declare that the research was conducted in the absence of any commercial or financial relationships that could be construed as a potential conflict of interest.
